# Perineal Pseudoaneurysm from Traumatic Foley Removal Leads to Recurrent Life-Threatening Hematuria

**DOI:** 10.1089/cren.2015.0009

**Published:** 2015-11-01

**Authors:** Lorraine Min-Shan Liang, Jingbing Xue, Erdal Erturk

**Affiliations:** ^1^ Department of Urology, University of Rochester Medical Center, Rochester, New York.; ^2^ Department of Imaging Sciences, University of Rochester Medical Center, Rochester, New York.

## Abstract

Hematuria resulting from urethral traumatic catheter insertion and removal is often encountered. Usually, hematuria resolves with conservative measures. We report a case of traumatic Foley removal leading to intermittent life-threatening hematuria resulting in blood loss anemia requiring multiple transfusions and multiple episodes of hypotension requiring pressors. A pelvic angiogram revealed a pseudoaneurysm of the left pudendal artery, which was treated with microcoil embolization leading to resolution of bleeding.

## Introduction and Background

Catheter-induced hematuria frequently occurs as a result of prostatic trauma, rapid bladder decompression, and creation of false passages.^1^ These usually resolve with conservative and supportive measures. We report a case of a pseudoaneurysm caused by traumatic Foley removal leading to recurrent life-threatening hemorrhage that ultimately required embolization for control of bleeding.

## Presentation of Case

A 40-year-old man with Down syndrome presents to the emergency department for increased seizure activity. He has a 16F Foley catheter with a 10 cc balloon placed that initially drains clear yellow urine. Twenty minutes later, the patient removed his Foley with the balloon inflated and hematuria ensues. The hematuria is initially managed with exchange to a larger sized Foley and frequent irrigations. His hematocrit fell from 45 to 21 over 2 to 3 days. He was considered a poor anesthetic risk, so conservative management was pursued for a prolonged period. He received blood transfusions but became hypotensive and required vasopressors. He was brought to the operating room for cystoscopy 5 days after initial injury. No active bleeding was identified from the bladder or prostate. There was a tear in the bulbous urethra with an adherent clot, but no active bleeding was seen. Due to his propensity for pulling out tubes and drains, decision was made to leave the patient without a Foley catheter.

Four days later, his gross hematuria recurred and his hematocrit fell to 20%. He received supportive care, including fluids, blood products, and bladder irrigation. A CT urogram only revealed a spongiosal tear. Over the next few days, his urine cleared and he became hemodynamically stable without vasopressors. On the 11th day after his initial injury, he had recurrence of gross blood from his urethra, which caused clogging of his catheter, a hematocrit drop from 28 to 20 and hypotension requiring pressors. He was brought to Interventional Radiology for angiography with a diagnostic and therapeutic intent.

**FIG. 1.**
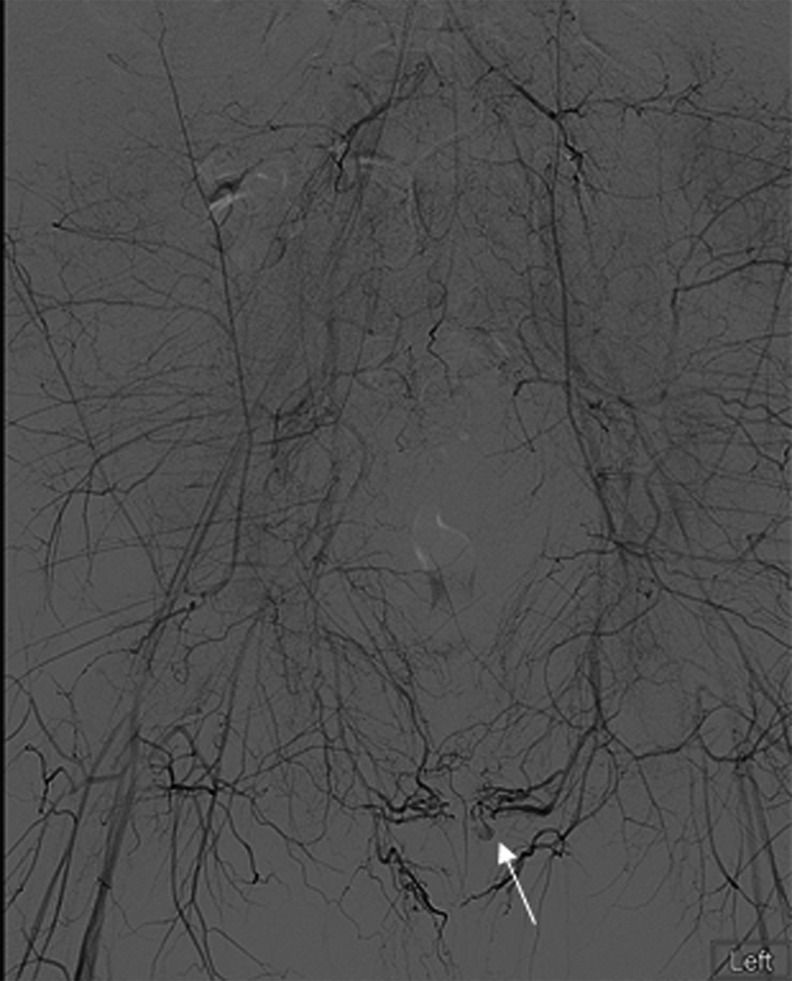
Aortogram with *arrow* pointing to pseudoaneurysm of the perineal branch of the left pudendal artery.

**FIG. 2.**
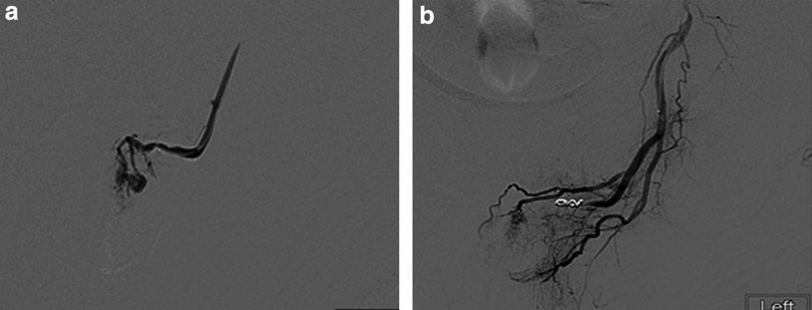
(**a)** Selective angiography demonstrating pseudoaneurysmal dilation of the perineal branch of the left pudendal artery. **(b)** Angiography shot after coiling of the pseudoaneurysm. This illustrates the lack of blood flow beyond the coil and no enhancement of the previously visualized pseudoaneurysm.

Selective pelvic angiogram (Figs. 1 and 2a) revealed a pseudoaneurysm of the perineal branch of the left internal pudendal artery. This was coiled using 3-mm microcoils (Fig. 2b).

**FIG. 3.**
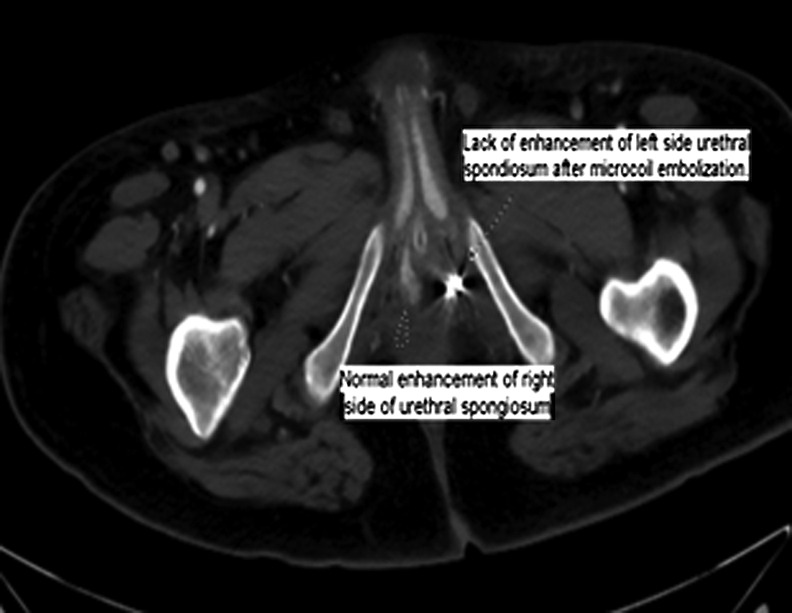
CT angiography demonstrates decreased enhancement of the left spongiosal tissue compared with the right spongiosal tissue after selective angioembolization.

Postoperatively, the patient's urine remained red, but hematocrit remained stable. The CT angiography (Fig. 3) revealed no evidence of bleeding. Urine cleared after irrigation removed old clot. The patient remained stable and, 1 week later, retrograde urethrogram revealed no evidence of urinary extravasation. The Foley catheter was removed and the patient was able to void and was discharged home.

## Discussion and Literature Review

Pseudoaneurysms are a dilation of two layers of the arterial wall resulting from adventitial injury. These may result from blunt force trauma, infection, or as a result of invasive procedures. Pseudoaneurysms lead to a pressurized dilation of the vessel leading to invasion of potential spaces in surrounding tissues.^1^ A pseudoaneurysm leading to life-threatening hematuria has been reported in one other case of catheter-induced trauma,^2^ but there are a handful of cases of lower urinary tract pseudoaneurysms in the urologic literature that have been effectively treated with angioembolization.^1,3,4^ In our case, the discovery of this pseudoaneurysm was unexpected but led to a good therapeutic solution.

## Conclusion

In situations where patients have delayed, intermittent, unexpectedly life-threatening hematuria and are not progressing and improving as expected, angiography and embolization are important diagnostic and therapeutic tools in the Urologists' arsenal.

## Abbreviation Used

CT: computed tomography
